# Diagnostic accuracy of cognitive screening tools under different neuropsychological definitions for poststroke cognitive impairment

**DOI:** 10.1002/brb3.1671

**Published:** 2020-07-03

**Authors:** Xiangliang Chen, Yunfei Han, Junshan Zhou, Minmin Ma, Xinfeng Liu

**Affiliations:** ^1^ Department of Neurology Nanjing First Hospital Nanjing Medical University Nanjing China; ^2^ Department of Neurology Jinling Hospital Medical School of Nanjing University Nanjing China

**Keywords:** neuropsychology, poststroke cognitive impairment, screening tool, telephone assessment

## Abstract

**Objectives:**

The accuracy of cognitive screening tools to detect poststroke cognitive impairment (PSCI) was investigated using various neuropsychological definitions.

**Methods:**

Hospital‐based stroke patients underwent a comprehensive neuropsychological assessment. The rate of PSCI was estimated using thresholds of 1, 1.5, or 2 standard deviations below the normal control and memory impairment defined by a single or multiple tests. Meanwhile, the diagnostic accuracy of cognitive screening through face‐to‐face assessment using the Mini‐Mental State Examination (MMSE) and the Montreal Cognitive Assessment Scale (MoCA), and telephone assessment using a 5‐minute NINDS‐Canadian Stroke Network (NINDS‐CSN) scale and a six‐item screener (SIS), was both tested under different definitions, with the optimal cutoff selected based on the highest Youden index.

**Results:**

In stroke patients, the rate of PSCI ranged from 46.3% to 76.3% upon different definitions. The face‐to‐face MoCA was more consistent with the comprehensive cognitive assessment compared to MMSE. The optimal cutoff of PSCI was MMSE ≤ 27 and MoCA ≤ 19. For the telephone tests, the 5‐minute NINDS‐CSN assessment was more reliable, and the optimal cutoff was ≤23, while for SIS ≤ 4.

**Conclusions:**

Cognitive screening tools including the face‐to‐face MMSE and MoCA, together with the telephone assessment of NINDS‐CSN 5‐minute protocol and SIS, were simple and effective for detecting PSCI in stroke patients. The corresponding threshold values for PSCI were 27 points, 19 points, 23 points, and 4 points.

## Graphical abstract

The rate of cognitive impairment varied as neuropsychological definitions differed. Cognitive screening cutoffs for MMSE, MoCA, NINDS‐CSN 5‐minute protocol, and SIS were 27, 19, 23, and 4.

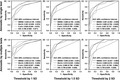

## 1 INTRODUCTION

Poststroke cognitive impairment (PSCI) is prevalent in over half of patients 6 months after stroke (Merriman et al., 2019), and cognitive assessment in stroke patients is essential. The standard process requires comprehensive assessment using well‐validated cognitive tasks (Gorelick et al., 2011). Our preliminary work has validated the NINDS‐CSN neuropsychological protocols (60, 30, and 5 minutes) in mild stroke patients (Chen, Wong, et al., 2015). However, there is no consensus on the definition of impairment in a particular domain, while PSCI is defined as deficits in at least one cognitive domain (Gorelick et al., 2011). For instance, the cutoff value would be set at 1, 1.5, or 2 standard deviations (*SD*) below the means of the healthy control, and it is inconsistent whether a single test or multiple tests should be considered when determining the impairment of a cognitive domain (Pendlebury, Mariz, Bull, Mehta, & Rothwell, 2013a). These would have affected the prevalence estimation of PSCI and hindered the comparison between various studies.

For time–cost optimization, cognitive screening tools have become the first choice in clinical practice, such as the Mini‐Mental State Examination (MMSE) and the Montreal Cognitive Assessment Test (MoCA; Ghafar, Miptah, & O'Caoimh, 2019). Moreover, telephone scales like the NINDS‐CSN 5‐minute protocol and the six‐item screener (SIS; Callahan, Unverzagt, Hui, Perkins, & Hendrie, 2002) are helpful for the large‐scale epidemiological survey and long‐term follow‐up studies (Levine et al., 2018). However, the thresholds used in each study are inconsistent, varying from 21 to 26, probably due to different diagnostic criteria, culture difference, and distinct education level of the subjects (Stolwyk, O'Neill, McKay, & Wong, 2014).

This study was based on the employment of different neuropsychological definitions in stroke patients. The rate and classification of PSCI were compared; meanwhile, the diagnostic accuracy and the optimal threshold of the screening tools MoCA, MMSE, the NINDS‐CSN 5‐minute protocol, and SIS were investigated.

## 2 METHODS

### 2.1 Subjects

Research subjects were stroke patients discharged from the hospital between August 2013 and June 2014 who fulfilled the following criteria: diagnosed with acute ischemic stroke at least 3 months ago, aged over 50 years old, and provided with voluntary informed consent to the cognitive assessment. Exclusion criteria were severe motor, speech, visual, or auditory impairment leading to inability to complete the tests, prestroke cognitive decline (Informant Questionnaire on Cognitive Decline in the Elderly, IQCODE ≥ 3.4), taking drugs that improve cognitive function, Parkinson's disease, intracerebral hemorrhage, and patients undergoing endovascular treatment. Recruitment details were described elsewhere (Chen, Fan, et al., 2015). The institutional review board at Jinling Hospital approved the study. All participants showed their voluntary agreement to take part in the study and signed the informed consent.

### 2.2 Comprehensive cognitive psychological assessment

The comprehensive cognitive psychology assessment was based on the 60‐minute NINDS‐CSN neuropsychological battery, including four cognitive domains: execution/attention (animal naming test, WAIS‐III Digit symbol‐coding test, Trail Making Test), language (modified Boston Naming Test), visuoconstruction (Rey‐Osterrieth Complex Figure Test [RCFT]‐copy trial), and memory (delayed recall on the revised Hopkins verbal learning test and RCFT recall test). It was tested after collecting the patient's clinical history. Impairment in at least one cognitive domain was required for the diagnosis of PSCI. Four subtypes were determined, including PSCI of amnestic single‐domain (only memory domain was impaired), amnestic multidomain (memory domain damaged, and at least one other cognitive domain was impaired), nonamnestic single domain (single domain other than memory was impaired), and nonamnestic multidomain (at least two cognitive domains other than memory were impaired; Gorelick et al., 2011).

These subtypes were distinguished under different definitions: (a) threshold of the test score using 1, 1.5, or 2 *SD* below the mean performance of healthy controls matched by age, sex, and education level in the previous study (Chen, Wong, et al., 2015), and (b) choosing one single test versus both tests of the memory domain to define amnestic PSCI. The rate and subtype of PSCI were compared within different definitions.

### 2.3 Cognitive screening test

Cognitive screening tests include face‐to‐face and telephone assessment. Screening tests of MMSE and MoCA Beijing version, together with the comprehensive cognitive assessment, were face‐to‐face measured with at least one hour apart. Mini‐Mental State Examination was tested at the beginning and MoCA tested at the end of the 60‐minute NINDS‐CSN scale. These patients were followed up with the telephone assessment, including the NINDS‐CSN 5‐minute protocol and the SIS scale. The NINDS‐CSN 5 minute protocol is a 30‐point scale with five subtests in MoCA‐BJ. The SIS is a 6‐point scale which includes the three‐word memory recall and three‐item temporal orientation components from the MMSE (Callahan et al., 2002). It was tested at first, followed by the 5‐minute protocol. The two telephone scales were completed by the same person (X. Chen) blinded to the results of the face‐to‐face assessment.

### 2.4 Statistical analysis

Data normality was tested using the Kolmogorov–Smirnov test. Data with a normal distribution were expressed as mean ± standard deviation, and those with a skewed distribution the median and interquartile range (IQR). The diagnostic accuracy of each cognitive screening scale was analyzed by the receiver operating characteristic (ROC) curve analyses with the area under the curve (AUC). If the AUC reached 0.7, the scale was considered to have sufficient accuracy to detect the cognitively impaired patients. The AUC between different scales was compared using the method described by Hanley and McNeil (Hanley & McNeil, 1983). The cutoff value for detecting PSCI was determined for each screening scale, and the optimal cutoff was established in terms of the highest Youden index. The threshold for the face‐to‐face screening scales was stratified with respect to education (Cui et al., 2011; Lu et al., 2011): illiterate/uneducated subjects: MMSE score 17/18 and MoCA score 13/14; subjects with 1–6 years of education: MMSE score 20/21 and MoCA score 19 /20 points; and subjects with an education of >6 years: MMSE score 24/25 and MoCA score 24/25. Meanwhile, the kappa statistic was used to evaluate the agreement of cognitive screening tests to the comprehensive cognitive assessment. A two‐sided *p* < .05 was considered to be statistically significant. All statistical analyses were performed using the SPSS 17.0 software package.

## 3 RESULTS

A total of 498 patients were screened during the study period, and 409 were excluded (258 had geographic limits, 36 lost contact, 10 had severe limb impairment, 2 had aphasia, 5 had visual or hearing impairment, 10 had prestroke cognitive decline or in use of cognitive enhancers, 2 had Parkinson's disease, 40 had a hemorrhagic stroke, 34 underwent stenting or balloon angioplasty, and there were 12 deaths before assessment). Hence, this study included 89 stroke patients with an average age of 62.9 ± 8.6 years, an education level of 9.2 ± 4.2 years, and a male ratio of 65.2%. The severity of stroke at admission was assessed using the National Institutes of Health Stroke Scale (NIHSS), and the median score for NIHSS was 2 points (IQR, 0.8–5). All patients underwent a comprehensive neuropsychological assessment and the face‐to‐face cognitive screening tests. The test was performed at 7.3 months (IQR, 4.8–9.4) after stroke onset. Eighty patients underwent the telephone tests, and the median interval between the face‐to‐face test and telephone assessment was 2.8 months (IQR, 1.7–7.0).

Among the 80 stroke patients who completed both the face‐to‐face tests and the telephone tests, the rate of PSCI was found to be 37/80 (46.3%) from the most conservative definition (2 *SD* below the control mean), to 61/80 (76.3%) as with the least conservative definition (1 *SD* below the control mean). The classification also varied. In general, there was a higher rate of patients classified as single‐domain impairment when more stringently defined. Nevertheless, those with amnestic single‐domain PSCI were rare regardless of the different definitions, accounting for only 0%–7% of the total number. In addition, the PSCI rate was slightly lower when using multiple tests to define memory‐domain impairment compared to using a single test, but the impact was not significant (Pearson's chi‐Square: 0.100–0.517, *p* range: .47–.75) (Table 1). On the other hand, despite the varied definitions, the PSCI patients reported more depressive symptoms as assessed by the geriatric depression scale (*p* range: .02–.11) with medium effect sizes (Cohen's *d*: 0.36–0.53), but they showed no differences for neuropsychiatric symptoms as assessed using the neuropsychiatric inventory questionnaire (*p* range: .12–.93).

**TABLE 1:** Effects of different neuropsychological definitions on the number of patients

Definition of amnestic^a^	Single test			Multiple tests		
Test thresholds^b^	1 *SD*	1.5 *SD*	2 *SD*	1 *SD*	1.5 *SD*	2 *SD*
Single‐domain PSCI						
Amnestic	4	2	2	0	0	0
Nonamnestic	7	6	10	15	16	15
Multiple‐domain PSCI						
Amnestic	45	34	22	18	12	8
Nonamnestic	5	4	5	24	16	14
Total PSCI, no. (%)	61 (76.3)	46 (57.5)	39 (48.8)	57 (71.3)	44 (55.0)	37 (46.3)

Abbreviations: NINDS‐CSN, NINDS‐Canadian Stroke Network; PSCI, poststroke cognitive impairment.

^a^ Amnestic PSCI was defined by either one or both memory tests in the 60‐minute NINDS‐CSN neuropsychological battery.

^b^ Test thresholds were selected below 1, 1.5, or 2 *SD* of the mean test scores of age, sex, and education matched healthy controls on the 60‐minute NINDS‐CSN neuropsychological battery.

**FIGURE 1:**
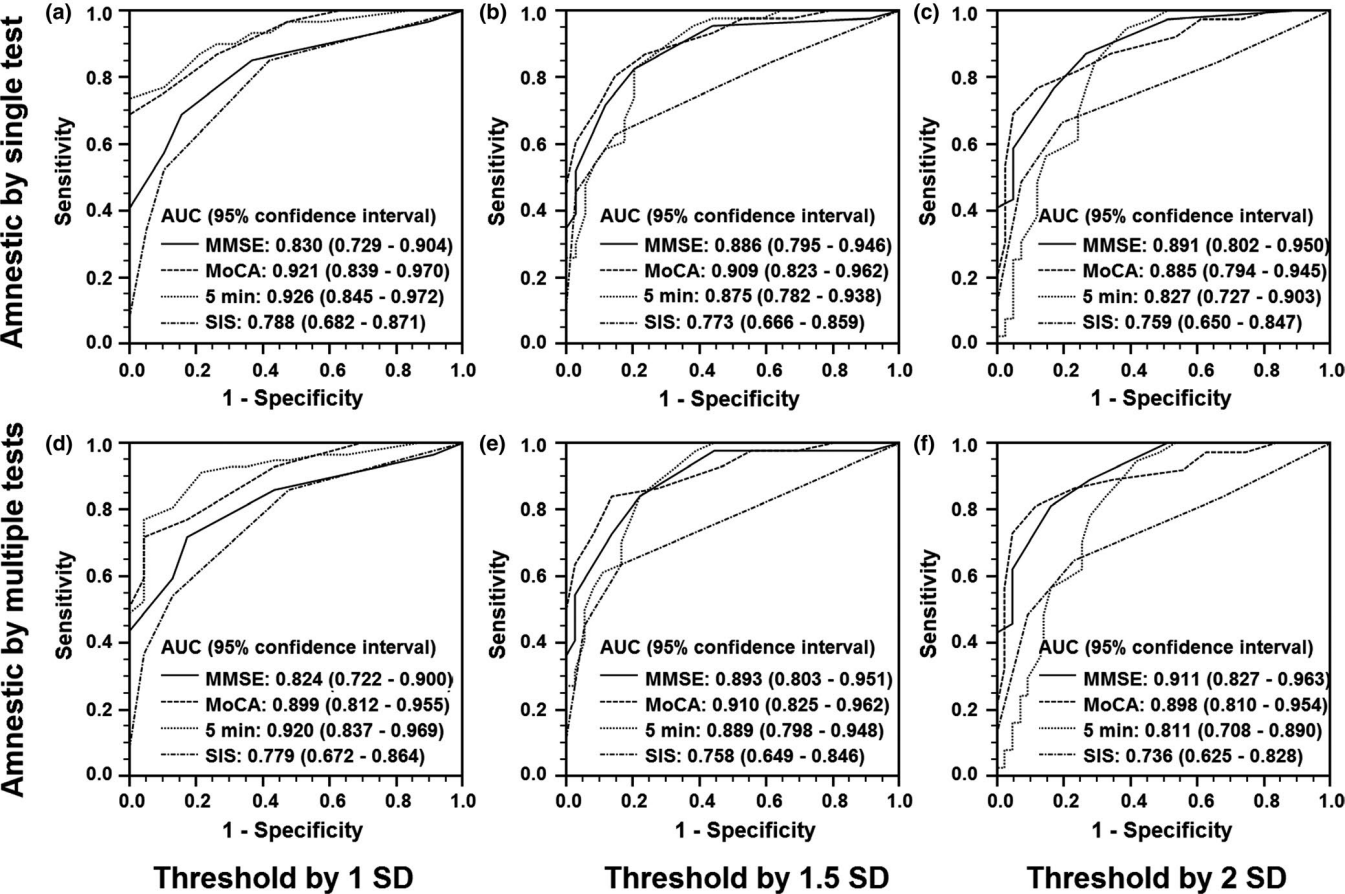
Receiver operating characteristic curves with area under the curve (AUC) values for the performance of face‐to‐face (MMSE and MoCA) and telephone (NINDS‐CSN 5 minute protocol and SIS) cognitive screeners under different neuropsychological definitions. MMSE, Mini‐Mental State Examination; MoCA, Montreal Cognitive Assessment Scale

The ROC curve analyses showed an AUC value above 0.7 for each cognitive screening scale despite different neuropsychological definitions, indicating good validity (Figure 1). The face‐to‐face screening tests of MMSE and MoCA had broadly similar AUC values. The two telephone screening tests were comparable for the 2 *SD* cutoff definition (Figure 1c,f). In comparison between the telephone and face‐to‐face tests, the SIS scale was generally less accurate than the MoCA (*p* range: .002–.04), while SIS was alternative to MMSE at 1 *SD* cutoff (Figure 1a,d, *p*: .61 and .55 based on single test and multi‐test, respectively). Besides, the NINDS‐CSN 5‐minute protocol was comparable to both face‐to‐face cognitive tests (*p* range: .12–.93) except for the most conservative definition at 2 *SD* cutoff and multi‐test required for memory impairment (Figure 1f, *p*: .05 and .03 compared to MMSE and MoCA, respectively).

The sensitivities and specificities of each scale were listed according to different neuropsychological criteria (Table 2), and the cutoffs to detect PSCI were determined. In most cases, sensitivity and specificity were optimal with MMSE ≤ 27 points, MoCA ≤ 19 points, NINDS‐CSN 5‐minute protocol ≤23 points, and SIS ≤ 4 points. The MMSE scores were skewed to the high scores, while the MoCA scores were normally distributed (*p* = .627 by a Kolmogorov–Smirnov test). Correlation analysis suggested that the two had a high correlation (Spearman's *r^2^* = .723; *p* < .0001). The cognitive screening scales with the optimal cutoffs reached a good agreement with the comprehensive cognitive assessment (*kappa*: 0.318–0.630 for the telephone scales, 0.411–0.697 for the face‐to‐face scales). There was a greater consistency for the NINDS‐CSN 5‐minute protocol than the SIS scale, and a greater consistency for MoCA than MMSE as well. However, based on the education‐stratified threshold, MMSE and MoCA were inconsistent with the comprehensive cognitive assessment (*Kappa*: 0.074–0.328).

**TABLE 2:** Cutoffs, sensitivity, and specificity of screening scales for cognitive impairment

Sensitivity, specificity (%)		Single test^a^			Multiple tests^b^		
Scales	Cutoffs	1 *SD*	1.5 *SD*	2 *SD*	1 1 *SD*	1.5 *SD*	2 *SD*
MMSE	25	41, 100	52, 97	59, 95	44, 100	55, 97	62, 95
	26	57, 89	72, 88	77, 83	60, 87	73, 86	81, 84^c^
	27	69, 84^c^	83, 79^c^	87, 73^c^	72, 83^c^	84, 78^c^	89, 72
	28	85, 63	96, 56	97, 49	86, 57	98, 56	100, 49
MoCA	18	57, 100	70, 91	77, 88^c^	60, 96	73, 92	81, 88^c^
	19	69, 100^c^	80, 85^c^	82, 76	72, 96^c^	84, 86^c^	86, 77
	20	75, 89	87, 76	87, 66	77, 83	86, 72	89, 65
	21	87, 74	93, 56	92, 46	88, 65	93, 53	92, 44
NINIDS 5 minutes	23	74, 100	83, 79^c^	85, 71	77, 96^c^	84, 78^c^	84, 67
	23.5	77, 89^c^	87, 74	90, 66	81, 87	89, 72	89, 63
	24	82, 84	91, 68	95, 61^c^	86, 83	93, 67	95, 58^c^
	24.5	87, 79	96, 62	97, 54	91, 78	98, 61	97, 51
SIS	3	34, 95	46, 97	49, 93	37, 96	45, 94	49, 91
	4	52, 89	63, 85^c^	67, 80^c^	54, 87^c^	64, 83^c^	65,77^c^
	5	85, 58^c^	85, 38	85, 34	86, 52	84, 36	84, 33

Note

Amnestic PSCI was defined by either ^a^one or ^b^both memory tests in the 60‐minute NINDS‐CSN neuropsychological battery. ^c^The highest Youden index under the classification.

Abbreviations: MMSE, Mini‐Mental State Examination; MoCA, Montreal Cognitive Assessment Scale; NINDS‐CSN, NINDS‐Canadian Stroke Network; PSCI, poststroke cognitive impairment; SIS, six‐item screener.

## 4 DISCUSSION

Our results suggested that under different neuropsychological definitions, face‐to‐face MMSE and MoCA, and telephone scales of NINDS‐5min protocol and SIS, were feasible and effective cognitive screening tools for stroke patients in China.

Previous studies have shown that the incidence rate of dementia and PSCI is affected by different diagnostic criteria (Pendlebury, Mariz, et al., 2013; Robertson et al., 2019). Our study indicated a lower rate of PSCI toward a more conservative definition, which was consistent with prior findings (Pendlebury, Mariz, et al., 2013). Besides, in line with clinical observations, stroke patients seldom suffered from amnestic single‐domain PSCI (Camarda et al., 2018). Moreover, the rate of patients with PSCI in multiple domains increased as the definition was less conservative. Such patients were reported to progress to dementia at a faster speed (Kim et al., 2019); hence, the differences in definition may affect the identification of individuals along the disease trajectory (Edmonds et al., 2019).

The study found that each cognitive screening scale can effectively distinguish stroke patients with PSCI regardless of various neuropsychological definitions. As a face‐to‐face test, either MoCA or MMSE can be applied to stroke patients in cognitive screening, and the former was more accurate in tracking mild cognitive impairment, though they were of the same effectiveness to detect dementia (Pinto et al., 2019). For telephone screening, the NINDS‐CSN 5‐minute protocol was a more effective scale than SIS in stroke patients (Chen, Fan, et al., 2015). Furthermore, a similar accuracy of the NINDS‐CSN 5‐minute telephone scale to both MMSE and MoCA was found in all cases except for the most conservative definition, and the accuracy of the SIS telephone scale was comparable to that of MMSE for the less conservative definition. Since a less conservative definition corresponds to a more sensitive screening, the NINDS‐CSN 5‐minute telephone scale can be utilized as an alternative to MoCA and MMSE, and SIS test to MMSE.

Both MMSE and MoCA are commonly used cognitive screening scales in stroke patients (Rodrigues et al., 2019). This study showed a ceiling effect of the MMSE, which was also reported in previous observations (Trzepacz, Hochstetler, Wang, Walker, & Saykin, 2015). On the other hand, scores of MoCA were normally distributed, and the consistency with comprehensive cognitive assessment was better than MMSE under different definitions. Therefore, MoCA may be more suitable than MMSE in the cognitive screening of stroke patients (Ghafar et al., 2019). It is noteworthy that according to a previous review, the 19/20 cutoff for MoCA‐BJ is at the lower bound of the optimal values ranging from 20 to 27 for cognitive normality in the chronic stroke phase (Chiti & Pantoni, 2014). Hence, there is a probability that PSCI would be false‐negative with this cutoff. Meanwhile, a previous report also showed a majority of false‐positive cases with the 21/22 cutoff in Chinese stroke patients (Wong et al., 2015). Therefore, appropriate precautions must be taken before using this statistically derived cutoff value to indicate a further comprehensive neuropsychological evaluation and to determine the potential clinical and functional importance.

In this study, more than half of the stroke patients were unable to participate in face‐to‐face cognitive tests because of geographical factors. Therefore, in studies of long‐term cognitive outcomes, patients would potentially benefit from telephone screening (Moffatt, Hennessy, Marshman, & Manickam, 2019). A recent UK community study firstly explored the feasibility of the NINDS‐CSN 5‐minute scale for telephone assessment and demonstrated sufficient reliability for cognitive screening 1 year after transient ischemic attack or mild stroke (Pendlebury, Welch, et al., 2013). The SIS Mandarin Scale has been proved efficient for face‐to‐face screening of cognitive impairment in the elderly (Xue et al., 2018) and was also used as a telephone tool for cognitive follow‐up in an American stroke study (Levine et al., 2018). Based on the preliminary validation of the NINDS‐CSN 5‐minute protocol and SIS as a telephone assessment tool in stroke populations, this study showed that under different definitions, the screening thresholds for PSCI were mostly ≤23 points and ≤4 points respectively, and the NINDS‐CSN 5‐minute protocol had a higher consistency than the SIS scale.

This study has the following limitations. First, this was a relatively small sample study with a majority of patients who underwent a mild stroke, and patients with right side hemiplegia, aphasia, and severe stroke were excluded. Second, we did not provide education‐based or age‐based cutoffs, and given that the average was comparatively well‐educated, and in the early sixties, the generalization would be limited to those who are illiterate, younger, or older patients (Wu, Wang, Ren, & Xu, 2013). Third, the scales were assessed cross‐sectionally, so their sensitivity to cognitive changes and cutoffs for cognitive decline remains worthy of further longitudinal investigations. Fourth, previous studies indicated progressive cognitive decline after stroke (Zheng, Yan, Zhong, Yang, & Xie, 2019), so the scores of telephone screeners, tested at 2.8 months after the face‐to‐face assessment, might be underestimated; however, this would possibly be neutralized by the practice effects associated with repeated testing items. Finally, cognitive screening tests were generalized rather than domain‐specific; hence, assessment of multiple cognitive domains and clinical examinations were still needed for clinical diagnosis.

## 5 CONCLUSION

This study provides preliminary evidence for use of two cognitive screeners applied in face‐to‐face interview (MMSE ≤ 27 points and MoCA ≤ 19 points) and another two that can be administered over the phone (NINDS‐CSN 5‐minute protocol ≤ 23 points and SIS ≤ 4 points) for detecting PSCI in stroke patients. The telephone cognitive screens may be of particular relevance in research when seeking patient eligibility to participate in trials. Our findings may facilitate further studies in terms of choice for cognitive screening tools under different neuropsychological definitions.

## CONFLICT OF INTEREST

The authors declare no conflict of interest.

## AUTHORS' CONTRIBUTIONS

Minmin Ma designed the study and supervised the data collection. Xiangliang Chen initiated the study and drafted the manuscript. Yunfei Han was involved in data collection, and he critically revised the manuscript. Junshan Zhou preformed the statistical analyses. Xinfeng Liu supervised the data collection and analyses.

## Data Availability

The data of this study are available upon reasonable request.
